# Multisensory signalling enhances pupil dilation

**DOI:** 10.1038/srep26188

**Published:** 2016-05-18

**Authors:** Silvia Rigato, Gerulf Rieger, Vincenzo Romei

**Affiliations:** 1 Centre for Brain Science, Department of Psychology, University of Essex, Colchester, CO4 3SQ, UK; 2 Social and Health Psychology, Department of Psychology University of Essex, Colchester, CO4 3SQ, UK

## Abstract

Detecting and integrating information across the senses is an advantageous mechanism to efficiently respond to the environment. In this study, a simple auditory-visual detection task was employed to test whether pupil dilation, generally associated with successful target detection, could be used as a reliable measure for studying multisensory integration processing in humans. We recorded reaction times and pupil dilation in response to a series of visual and auditory stimuli, which were presented either alone or in combination. The results indicated faster reaction times and larger pupil diameter to the presentation of combined auditory and visual stimuli than the same stimuli when presented in isolation. Moreover, the responses to the multisensory condition exceeded the linear summation of the responses obtained in each unimodal condition. Importantly, faster reaction times corresponded to larger pupil dilation, suggesting that also the latter can be a reliable measure of multisensory processes. This study will serve as a foundation for the investigation of auditory-visual integration in populations where simple reaction times cannot be collected, such as developmental and clinical populations.

Perceiving the environment in a coherent way largely depends upon an ability to integrate information across multiple senses. Multisensory integration reduces perceptual ambiguity^1^ and enhances the detection of stimuli in space and time^2^. Behavioural studies recording manual reaction times (RTs) have revealed that participants are systematically faster at responding when an auditory and a visual stimulus are simultaneously presented compared to when they are presented alone (e.g.^3^). However manual RTs cannot always be recorded; for example this might not be possible in certain developmental and clinical populations. In the current study, we sought to overcome this limitation by exploring a novel way for measuring auditory-visual integration via pupil dilation.

The measure of pupil size is a non-invasive indicator of reactions that occur spontaneously during stimulus presentation, do not require overt responses^4^,^5^, and can be reliably observed even in young infants^6^, clinical populations^7^,^8^, and non-human primates^9^. The pupil automatically dilates with sympathetic activity and constricts with parasympathetic activity (see^10^). Pupil dilation is controlled by brain areas associated with both cognitive and emotional processing (e.g.^11^), suggesting that it may represent a summative index of brain activity associated with performing cognitive and emotional tasks. Pupils dilate more strongly when higher attentional allocation is requested, or during memory tasks, interpretation of more difficult material or during target detection tasks (for review, see^12^,^13^,^14^). Further, when cognitive demands triggered by tasks or stimuli are continuous, pupils remain dilated^12^. Such dilation is likely to be associated with brain regions that are responsible for sustained attention mechanisms and on-going processing of information^15^,^16^.

Of particular interest for the study of multisensory integration is the recent evidence that the superior colliculus (SC) may play a further role in coordinating responses towards salient stimuli, including transient pupil responses^9^. The SC is a midbrain structure that receives inputs from the retina and visual cortex at its superficial layers, but also multisensory inputs at its intermediate layers^2^,^17^. Evidence in favour of a SC role in pupil responses comes from animal studies, both with mammals^9^,^18^ and birds^19^. For example, Wang and colleagues^9^ examined pupil responses in rhesus monkeys following the presentation of visual, auditory, or auditory-visual stimuli. The pupil responded similarly to visual and auditory stimuli, and the responses to the bimodal stimuli were well approximated by the linear sum of the responses evoked by the stimulus components. In a recent review, Wang and Munoz^20^ also showed human responses evoked by auditory, visual, and auditory-visual stimuli. In contrast with the animal study, they observed in humans larger and more sustained pupil dilation to the multisensory stimuli than each of the unimodal conditions^21^ (Conference Proceedings). However, to the best of our knowledge there is not reported information about analysis that addresses the superadditivity criterion as we present in this paper.

Despite these recent advances, it has yet to be demonstrated whether pupil dilation may represent a reliable index of multisensory integration that reflects established patterns of integration across the senses as assessed by other measures, such as RTs. For this purpose, we collected pupil responses and behavioural RTs, to auditory, visual and auditory-visual stimuli (Fig. 1). We tested for multisensory integration by applying the *race model inequality*^22^ to the RTs distribution and the *superadditivity* criterion to the pupil dilation response to examine whether responses to the multisensory stimuli would exceed the sum of the responses evoked by the auditory and visual stimuli alone. Firstly, we anticipated replicating the findings of the *race model inequality* for the RTs (e.g.^3^,^23^). Further, if pupillary responses are modulated by multisensory processes, a similar *superadditivity* effect would also be predicted. Finally, if both pupil changes and RTs index multisensory processing, a negative correlation between these two measures would be expected.

## Results

### Reaction times

Mean RTs to auditory (A), visual (V), and auditory-visual (AV) multisensory stimuli are shown in Fig. 2A. A repeated measures ANOVA revealed a significant main effect of stimulus condition, *F*(2, 134) = 101.739; *p* < 0.0001; η^2^ = 0.60. Follow-up contrasts confirmed that this effect was attributable to the facilitation of RTs in the multisensory condition (mean = 362.33 ms) versus either V (mean = 423.22 ms; *t*(67) = 10.2; *p* < 0.0001; Cohen’s *d* = 0.55) or A (mean = 449.07 ms; *t*(67) = 15.98; *p* < 0.0001; Cohen’s *d* = 0.76) conditions. Reaction times to A and V unimodal stimuli also differed, *t*(67) = −3.59; *p* = 0.001; Cohen’s *d* = 0.23. Additionally, there was violation of the *race model inequality* over the fastest 40% of the RT distribution (Fig. 2B,C). This violation is indicative of a redundant signals effect exceeding predictions based on simple probability summation, and therefore suggests multisensory integration. These findings thus replicate previously observed effects^3^,^21^,^22^,^23^,^24^,^25^,^26^ with an overall RT facilitation of 60.89 ms and 67 out of 68 participants showing such effect.

### Pupil data

In the next analyses we examined pupil dilation as a function of time for the three conditions (Table 1). Multiple regression analyses included participants as a random effect. For the condition with AV stimuli, results suggested a significant linear effect of time on pupil dilation, *p* < 0.0001 *β* = 0.14, which was qualified by a significant cubic effect *p* < 0.0001 *β* = −0.07. Other higher order effects were not significant and are not reported in the text but are listed in Table 1. For the condition with A stimuli, there was a significant linear effect of time on pupil dilation, *p* < 0.0001 *β* = 0.06, which was qualified by a significant cubic effect, *p* = 0.01 *β* = −0.02. Finally, for V stimuli, there was a significant linear effect, *p* < 0.0001 *β* = 0.06, which was qualified a by significant cubic effect, *p* < 0.0001 *β* = −0.07. Given the direction and strength of the linear effects, these analyses suggested that, independent of any curvilinear effects, the simple effect of time on pupil dilation was, in general, stronger for AV stimuli than for A stimuli and V stimuli (Fig. 3A). All of these effects are corrected for significant autocorrelations that were detected within participants.

Mixed factorial regression analyses confirmed these differences in pupil dilation to the different types of stimuli. These analyses accounted for repeated entries of participants across stimuli and conditions (by including participants as a random effect), and were corrected for autocorrelations of data. Analyses included interactions of stimulus type and time, predicting pupil dilation. With these interactions we computed whether the relationship of pupil dilation and time was significantly stronger for one stimulus condition than another. The linear increase of dilation over time in the AV condition as compared to the A condition was significant, *p* < 0.0001 *β* = 0.04. Simultaneously, the cubic effect of time on pupil dilation was significantly stronger for AV than for A, *p* = 0.0001 *β* = 0.02. For the comparison of AV with V, the liner effect of time on dilation was stronger for AV than V, *p* < 0.0001 *β* = 0.04. For the comparison of A and V conditions, a significant difference in the cubic effect of time on dilation was detected, *p* = 0.007 *β* = 0.02, which was explained by a stronger increase in dilation midway during stimuli presentation in the A condition, as compared to the V condition (Fig. 3A).

An alternative way to illustrate these differences is to average data points for each time frame across participants and regress these aggregated dilation scores against time. Albeit more crude than previous analyses, such procedure allows depicting aggregated data points in comparison to the fitted regression slopes. Figure 3B shows that, overall, there were differences in the curvilinear (e.g. quartic) relationship between time and pupil dilation that are very similar to those previously reported. Dilation in response to the AV condition became stronger as compared to both baseline and to each unisensory stimulus. The overall effect of time on dilation was strongest for AV stimuli, followed by A and V stimuli, *p* < 0.0001, *R*^*2*^ = 0.82, *p* < 0.0001, *R*^*2*^ = 0.54, and, *p* < 0.0001, *R*^*2*^ = 0.31, respectively.

In order to identify the time onset and precise temporal profile of the multisensory effect, we used a Monte Carlo simulation method over a temporal window between 1000 and 2000 ms, on a sample-point basis. Response to AV starts to differ reliably from A and V at around 1200 ms and in several discrete time windows until the end of the trial window (the simulation identified any sequence of consecutive significant *t* tests longer than 16.65 ms to be reliable). Figure 3 indicates the critical period in which A and V show the strongest difference compared to AV (red shadow depicts the difference between AV and the average of A and V).

Further to these analyses, the *superadditivity* criterion was employed to test whether pupil changes to multisensory stimuli exceeded the linear sum of the responses evoked by the A and V stimuli alone, [AV > A + V]. The one-tailed *t* test reached significance when the pupil data was averaged over the full 2 s period of stimulus presentation, *t*(67) = 2.134, *p* = 0.019; Cohen’s *d* = 0.23, and also when the analysis was restricted to the 1000–2000 ms time window for maximum dilation, *t*(67) = 2.134, *p* = 0.018; Cohen’s *d* = 0.23, confirming that the linear sum of the two unimodal conditions is less than the pupil changes obtained in the multisensory condition. Forty-seven out of 68 participants showed enhanced pupil dilation in response to multisensory compared to unisensory stimuli.

### Correlation

Finally, a Pearson correlation (2-tailed) between the RTs and the pupil dilation values for the AV condition showed a significant negative correlation when the pupil data was averaged over the full 2 s period of stimulus presentation, *R* = −0.317, *p* = 0.008, and also when the analysis was restricted to the 1000–2000 ms time window for maximum dilation, *R* = −0.297, *p* = 0.014; the faster the RTs, the greater the pupil dilation (Fig. 4). No such correlation was found for the V (*R* = −0.074, *p* = 0.548) and A conditions (*R* = −0.191, *p* = 0.118).

## Discussion

Research in multisensory integration has largely demonstrated that manual reaction times are a simple and reliable measure for studying this process (e.g.^3^,^23^). However, this measure cannot be applied in all contexts. In the current study, we explored pupillometry as an alternative method for the investigation of multisensory processes. For this purpose, we first adapted a simple RT paradigm, which we showed to be a reliable measure of multisensory integration. The analyses of the RTs confirmed facilitation in the multisensory condition, and a violation of the *race model inequality*^22^ suggested multisensory integration. Subsequently, we employed this paradigm to collect pupil responses to auditory, visual and auditory-visual stimuli. Pupillary responses to multisensory stimuli exceeded those to single modalities. In addition, pupil changes to auditory-visual stimuli exceeded the sum of the responses evoked by the unimodal stimuli, as tested for the *superadditivity* criterion. Finally, a negative correlation between RTs and pupil dilation to auditory-visual stimuli was found, which suggests that pupillometry can successfully be used to investigate multisensory integration processes.

In line with our findings, recent work by Wang and Munoz^20^,^21^ reported human pupil responses evoked by A, V and AV stimuli. Similar to our findings, A stimuli evoked a sustained enhancement of pupil size, while V rather evoked reduction in pupil size. Importantly, the larger and more sustained pupil dilation obtained in response to the multisensory stimuli compared with the unimodal conditions appears to resemble the magnitude of the effects observed in our own data, although a superadditivity analysis has not been specifically reported^20^ (Fig. 3B). Our experimental protocol was designed to provide an account of superadditivity by directly testing this hypothesis. Specifically, this was achieved by using low contrast visual stimuli and weak auditory stimuli, known to enhance multisensory gain (inverse effectiveness rule)^2^.

How are measures of pupil dilation and multisensory processes linked? Recently, microstimulation studies in animals have provided evidence for a role of the superior colliculus (SC) in pupil dynamics^27^,^28^. Specifically, the SC has been shown to play a role in encoding stimulus saliency to coordinate responses to stimuli, including pupil dilation^9^,^18^. At the same time, there is ample evidence of the central role played by the SC in multisensory processing^2^. As a consequence, our findings could be explained i) as a result of multisensory integration; ii) as a saliency effect; iii) as a combination of these two factors, i.e. the multisensory benefit is driven by the intrinsic saliency carried by multisensory stimuli.

According to the first interpretation, the greater pupil dilation evoked by the combination of auditory and visual stimuli compared to the pupil changes in response to the unimodal stimuli is obtained as a result of multisensory integration already at the level of the SC. This, in turn, would affect pupil dilation through modulation of sympathetic activity.

It may be alternatively conceived that our findings are better explained by a saliency effect due to the simultaneous presentation of two stimuli in different modalities. In line with this, such effect could have been in principle achieved even through redundant presentation of stimuli within the same modality - which would be more salient than a single stimulus. However this explanation seems unlikely based on the following empirical evidence: i) stimuli quickly presented in succession within the same modality are better explained by a linear summation over time of the same stimuli^29^; ii) when multisensory stimuli are simultaneously presented but mis-located, a linear effect best explains the pupil response to the stimulation^9^. In fact, in order for multisensory integration to take place, the visual and the auditory stimuli must not only be presented at approximately the same time (as stated in the temporal rule), but should also be perceived as coming from the same location (as stated in the spatial rule)^2^. The spatial rule can then explain the discrepancies between our findings and those of a previous study conducted with rhesus monkeys^9^. In this case the two unisensory stimuli were mis-located, and the results indicated that pupil responses to bimodal stimuli were well approximated by the linear sum of the responses evoked by the stimulus components. In contrast to this, our data cannot be explained by a simple linear summation and, in fact, they fit a *superadditivity* criterion.

Finally, it may be the case that both interpretations above are not mutually exclusive, i.e., our findings can be regarded as a multisensory advantage driven by the intrinsic saliency carried by multisensory stimuli, which may automatically attract attention. Indeed, previous research has shown that modulation of pupil dilation has been ascribed to cognitive and attentional load mechanisms among others (for a review, see^12^). In this respect, multisensory signals may naturally represent more salient stimuli than simple unisensory ones (e.g.^30^,^31^), thus being particularly effective in activating the anatomo-functional pathway responsible for the pupil dilation modulation. Greater pupil dilation is also associated with visual detection, and it occurs even when viewers do not overtly report seeing the targets^14^. Dilation of the pupil may therefore represent an adaptive response which serves the functional advantage of detecting the most significant stimuli in the environment.

To our best knowledge, this is the first evidence that pupil dilation can be an index of auditory-visual integration in humans, and bears relevance not only for the investigation of multisensory processes, but also for methodological applications. Despite the recent advances in psychophysiological and neuroimaging methods, such as EEG, fMRI or NIRS, and the vast use of these techniques in several populations, there are still limitations in terms of easiness of application and attrition. For example, in developmental studies, the attrition rate increases with age, from a relatively low attrition in infants (0–12%), to 30–45% in preschool children (for a review, see^32^). fMRI studies with adult clinical populations are also challenging and attrition can be as high as 60% with bipolar patients (e.g.^33^). We identified in pupillometry an alternative method for the investigation of multisensory processes (for implementation of other methods in the assessment of multisensory processes, see^34^), as it is a non-invasive methodology that does not involve sophisticated equipment preparation - if not a quick calibration task before the actual stimuli presentation. This is essential when testing populations, who, for example, might have a short attention span, and where simply adding additional time to experimental procedures can in fact jeopardise the chances of collecting reliable data. Further, collecting pupil data only requires passive stimulus processing as pupil changes occur automatically and spontaneously (e.g.^5^).

However, it is important to highlight the limitations of this measure. In our sample an effect of multisensory advantage as measured through pupil dilation could be found for 47 out of 68 participants. In contrast, such multisensory advantage could be found for RTs in 67 out of 68 participants, suggesting that pupil dilation represents a gross and indirect measure of multisensory integration compared to RTs (i.e. requiring activation of slower pathways through sympathetic and parasympathetic systems). Particularly, given the inter-subject variability in pupil dilation, it may prove difficult to identify a reliable effect at the individual level. Yet, our study demonstrates that such measurement at the group level is a reliable index of multisensory integration in at least two ways. We showed that, on average, AV conditions led to larger pupil dilation than A and V conditions, and that this effect was higher than the linear sum of A and V. Furthermore, we noted that despite the weak pupil responses evoked by V stimuli when presented in isolation the amplitude of the responses to the combination of A and V was enhanced, as predicted by the principle of inverse effectiveness^2^. Finally, such effect showed a negative correlation with a well-established measure of multisensory integration (i.e. RTs). In other words, subjects who were faster also showed stronger pupil responses in the multisensory condition. Can this be explained best by a specific effect of the multisensory advantage per se in both measures? It could be argued that alternative mechanisms may account for this correlation. For example, interindividual differences in task engagement could in principle explain the link we observed between these two measures, independently of multisensory processes. However, according to this explanation, it would be expected that the correlation should hold true independently of the stimulus type. However, we find this correlation for AV but not for the unisensory stimuli, thus supporting a multisensory account. Still, it could be argued that multisensory stimuli represent salient cues able to prompt more effective engagement strategies than unisensory stimuli. While we cannot readily rule out this alternative interpretation, these two accounts may be not mutually exclusive. As already argued above, multisensory stimuli may intrinsically convey more salient information^30^ which in turn tap into attentional mechanisms where interindividual task engagement may play a role. Future studies will be able to shed light on this phenomenon and better define its significance and interpretation.

In conclusion, these findings suggest that pupillometry might provide us with a novel measure to study multisensory integration processes, and future studies could use pupil dilation in populations where simple RTs cannot be collected. For example, pupil dilation can be used to identify the origin and to trace the development of multisensory integration early in infancy as well as for the study of multisensory impairments in developmental disorders (e.g.^35^).

## Methods

### Participants

Sixty-eight volunteers recruited at the University of Essex took part in this study (41 females; mean age 22.7 years, SD = 6.7). All participants had normal hearing and normal or corrected-to-normal vision. They all gave written informed consent to the study, which was approved by the Ethical Committee of the Department of Psychology, University of Essex. The methods were carried out in accordance with the approved guidelines. An additional 12 subjects were tested but their data were excluded from the analyses due to technical failure of pupil data recordings.

### Stimuli

**Figure 1:**
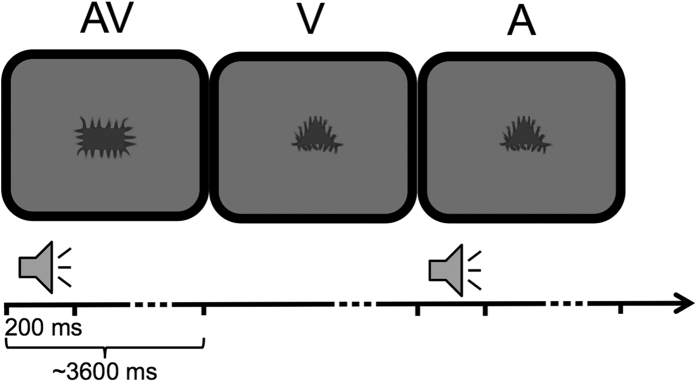
Schematic representation of the 3 types of trials (V, A, AV). In AV trials a novel visual stimulus appears on the screen in combination with an auditory stimulus. In V trials a novel visual stimulus alone appears on the screen. In A trials a tone is played while the visual image does not change from the previous trial. Within each trial, lasting ~3600 ms, visual stimuli are continuously displayed while auditory stimuli (in A and AV trials) are played for 200 ms.

**Figure 2:**
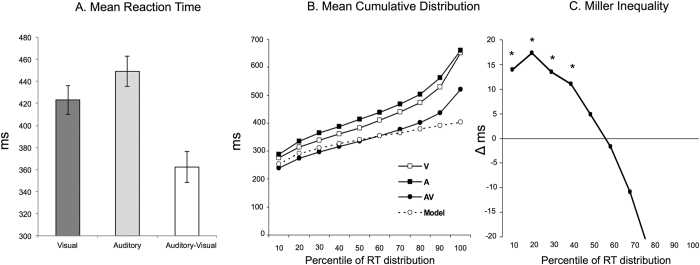
(**A**) Mean RTs (ms) to V, A, and AV stimuli. (**B**) Group averaged cumulative distribution for each stimulus condition and Miller’s^22^ model predictions. (**C**) RTs to AV stimuli exceeded prediction of probability summation over the fastest part of the distribution (indicated by positive values). *p < 0.05.

Task-relevant sensory events consisted of auditory (A), visual (V), or simultaneous auditory-visual (AV) multisensory stimuli. The visual stimuli were 9 greyscale abstract shapes (on average 10 × 6 cm) centrally presented on a darker grey background on a Tobii X-300 eye tracking monitor (70 cm viewing distance) (Fig. 1). Abstract neutral shapes were chosen as stimuli to ensure that any dilation occurred mostly to changes in visual information, avoiding the contribution of emotional information^36^,^37^. The auditory stimuli were 3 pure sinusoidal tones, 44,100 kHz sampling rate (200 Hz, 500 Hz, 800 Hz), presented through the monitor loudspeakers placed on each side of the screen, so that they were perceived at the midline of the visual field, and thus co-localized with the visual stimulus.

### Procedure

Participants were seated in a comfortable chair in a dimly lit room. Each participant completed two experimental blocks, each block containing 54 trials per condition (i.e. 162 trials in total). We have adapted the stimulus presentation parameters of a standard behavioural paradigm generally used for the study of auditory-visual integration (e.g.^3^,^23^) in order to minimise its impact on pupil dilation responses under unisensory conditions. To avoid saturation of pupil response^38^ we used low contrast visual and low intensity auditory stimuli (see Fig. 1). To avoid abrupt change in visual stimulus contrast the paradigm did not include an inter-trial fixation point and instead a visual stimulus (i.e. an abstract shape) was continuously displayed throughout the experiment. This way the impact of visual stimuli alone on pupil size would be reduced, thus making the paradigm well suited to test multisensory integration by exploiting the inverse effectiveness rule^2^.

In V conditions, a visual stimulus was presented alone throughout the trial and the start of the next trial corresponded to the presentation of a new visual stimulus. In A conditions, visual stimuli remained displayed on the screen from the previous trial, while a tone was played for 200 ms, at the onset of the new trial. In AV conditions, a new trial started with the presentation of a new visual stimulus paired with a tone. Stimuli from the 3 conditions were presented at different inter-stimulus intervals (with each trial lasting 3 s plus a random period lasting between 200 and 1000 ms) so as to avoid predictability and response anticipation. The same stimuli were used in the two blocks, however they were randomly selected each time. In the first block participants were instructed to make a button press on a keypad with their right index finger as quickly as possible after A, V or AV stimulus detection, while maintaining fixation on the centrally presented stimulus. Their manual RTs were recorded with E-Prime 2.0.

In the second block, the modulation of their pupil dilation by the sensory stimuli was recorded with an infrared eye tracker (Tobii X300, 1920 × 1080 pixel resolution) during passive exposure to the presented stimuli. A 5-point calibration of the eye-tracker took place before the block started; participants were asked to look at a dot moving on the screen while keeping their head still. Participants were then instructed to sit still in front of the monitor while looking at the screen and paying attention to the stimuli, and to avoid head and body movements. The two dependent variables (RTs and pupil dilation) were collected in separate blocks because RTs’ assessment necessarily required participants to move their hands (and as a result their whole upper body), whereas for the assessment of pupil dilation it is crucial that participants sit still without moving their bodies in order to avoid contamination of pupil dilation by movement-induced artefacts and spurious contribution of movement to pupil dilation^39^. Finally, recording RTs and pupil dilation in separate blocks allowed us excluding the confound of unbalanced motor contribution to the evaluation of pupil responses to multisensory stimuli (one motor response) and to the sum of unisensory stimuli (two motor responses) (see^40^ and data analysis below). Importantly, separate measurements of RTs and pupil size served the purpose of testing the impact of multisensory integration on pupil size without the potential influence of motor planning as would be the case for populations where RTs cannot be recorded.

### Measures and Analyses

#### Reaction times

Trials with RTs faster than 100 ms were not considered in the analysis to exclude outliers associated with stimulus anticipation. This fixed cut-off was chosen in accordance with previous studies using simple reaction time paradigms^3^,^41^,^42^,^43^. To verify that multisensory stimulus presentation facilitated reaction times relative to either unimodal condition, data were submitted to a repeated-measure ANOVA using stimulus condition (A, V, AV) as the within-subject factor. Faster reaction times to the multisensory condition would be evidence of a redundant signals effect. Moreover, we performed an analysis using Miller’s *race-model inequality*^22^, which assesses whether reaction time facilitation exceeds predictions of probability summation. To analyse the *race model inequality*, we used RMI test software (http://psy.otago.ac.nz/miller/Software.htm#RMITest), which implements the algorithm described in Ulrich *et al.*^44^. Briefly, the procedure requires four steps. First, for each condition (i.e. A, V and AV), participants’ RTs are converted to cumulative distribution functions (CDFs). Second, the race model distribution is calculated by summing the CDFs of the responses to the two unimodal conditions to create a “predicted” multisensory distribution. Third, percentile values (i.e. 5th, 15th, 25th, 35th, 45th, 55th, 65th, 75th, 85th, and 95th) are determined for every distribution of RT. Finally, the summary measure for each percentile value of each condition is calculated by determining the average of that percentile value across participants. The mean RT for the multisensory condition and the “predicted” condition are then compared for each percentile using a *t* test. Typically, the race model is rejected as insufficient to account for the data if there is a significant violation at any percentile, i.e. if significant values are obtained in the multisensory condition relative to the “predicted” condition. In this case, we would therefore conclude in favour of the existence of an integrative process.

#### Pupil data

A Tobii eye tracker recorded pupil data from each eye with a sampling frequency of 300 Hz, where each sample point corresponded to 3.33 ms. We recorded 750 samples for each trial, including a baseline period before stimulus onset (150 samples, corresponding to 500 ms) and the stimulus presentation (600 samples, corresponding to 2 s). The collected measure was pupil diameter calculated from the reflecting light of the pupil hitting the lens. To control for any head movement, the measure has been corrected for the distance to the lens at any sample point. In all subsequent analyses, we averaged pupil data from both eyes at each sample point. Finally, the entire period following stimulus onset has been baseline corrected to control for the variability in pupil diameter between participants, similar to previous reports (e.g.^6^,^45^,^46^). Data points lost due to eye blinks and movements have been coded by the system with an arbitrary –1 value. These values were excluded from all analyses.

Following data pre-processing, we examined how strongly pupils dilated over time to the AV condition, as compared to the single modalities, on average. For each type of condition (A, V, AV) we regressed pupil dilation against time in order to examine changing dilation patterns over time. Repeated measures within participants across stimulus conditions and time were accounted for by including participants as a random effect, i.e. for each type of stimulus, effect estimates (slopes) of time on pupillary response were first calculated within participants and then averaged across participants. Because it was possible that the correspondence of pupil dilation and time was not linear (e.g.^5^), we included higher order effects (i.e. quadratic, cubic and quartic effects) in these analyses. Hence, we tested whether the effect estimates of the averaged linear effects of time on pupil dilation differed significantly from the null hypothesis (which expects no slope), and simultaneously, whether the effect estimates of the averaged quadratic effect differed significantly from the linear effect, whether the effect estimates of the averaged cubic effect differed significantly from the quadratic effect, and so forth. Analyses were corrected for autocorrelation of data.

**Figure 3:**
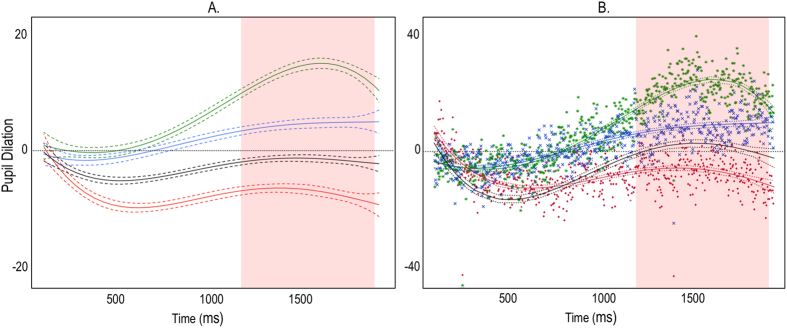
Pupil dilation and time since stimulus onset for AV (green), A (blue), and V (red). In addition, the black line indicates the expected linear summation of A and V. The Y-axis shows dilation as compared to baseline. The X-axis shows time elapsed since stimulus onset in ms. Lines represent regression coefficients with their 95% confidence intervals. The red shadow depicts the time-window where statistically reliable difference between AV and the average of A and V is found, i.e. between 1255 and 1948 ms. **3A.** Regression coefficients are computed by averaging individual coefficients across participants. **3B.** Regression coefficients are computed by averaging dilation scores across participants for each timeframe and regressing them on time. Dots represent averaged dilation scores. The scale on the y-axis is set to be twice as large for Fig. 3B than 3A in order to show the full range of effects in Fig. 3B. This reflects that effects are generally larger in Fig. 3B than 3A.

**Figure 4:**
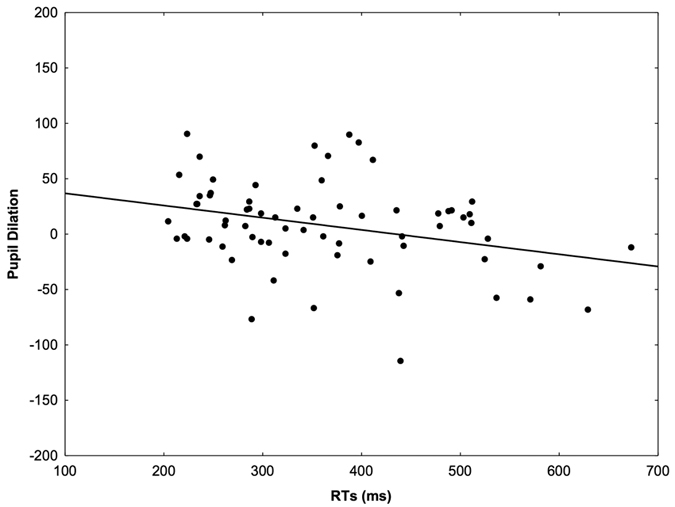
Significant correlation between the RTs and the pupil dilation values for the AV condition, indicating that shorter RT values correspond to greater pupil changes.

We further compared the stimulus conditions using paired *t* tests at each sample point specifically between 1000 and 2000 ms following stimulus onset. In this way, we compared reactions over a time-window for which strong dilation in humans, especially to AV, has been previously reported^20^ (Fig. 3B), and which excludes potential pupil constriction due to stimulus onset within the first 1000 ms^47^. A sample-point by sample-point analysis was carried out in order to establish the precise onset of the effects and to determine the time course over which the pupil changes evoked by the stimulus conditions differed. We corrected for the autocorrelation of consecutive sample points by using a Monte Carlo simulation method based on Guthrie and Buchwald^48^ (see^49^,^50^). This method began by estimating the average first order autocorrelation present in the real difference waveforms across the temporal window noted above. Next, 1000 datasets of randomly generated waveforms were simulated, each waveform having zero mean and unit variance at each time point, but having the same level of autocorrelation as seen on average in the observed data. Each simulated dataset also had the same number of participants and time-samples as in the real data. Two-tailed one sample *t* tests (vs. zero; alpha = 0.05, uncorrected) were applied to the simulated data at each time point, recording significant vs. non-significant outcomes. In each of the 1000 simulations the longest sequence of consecutive significant *t* test outcomes was computed. The 95th percentile of that simulated distribution of “longest sequence lengths” was then used to determine a significant difference waveform in the real data; specifically, we noted any sequences of significant *t* tests in our real data which exceeded this 95th percentile value. This method thus avoids the difficulties associated with multiple comparisons and preserves the type 1 error rate at 0.05 for each difference waveform analysed.

Finally, the *superadditivit*y criterion was employed to test whether pupil changes to multisensory stimuli exceeded the linear sum of the responses evoked by the auditory and visual stimulus alone [AV > A + V]. This pattern would indicate effective multisensory integration, as such activation cannot be accounted for through independent unisensory processing^51^. This analysis was carried out over the entire trial duration, i.e. 2 s after stimulus onset, and repeated over a shorter period as in previous analyses (between 1 and 2 s) where the AV effect is maximal.

When either redundant visual or auditory stimuli are presented, the output is a linear combination of two identical pupil responses^29^. However a linear summation of two unimodal stimuli might not account for simultaneous presentation of auditory and visual stimuli. Instead, a *superadditivity* model may better fit this multisensory process. To directly test this assumption, the sum of the pupil data averaged across time for the auditory and visual conditions was first calculated, and then compared with the pupil data collected in the auditory-visual condition averaged across time by means of one-tailed *t* test. This model is generally used in M/EEG data to detect nonlinear neural responses to AV stimuli and repeatedly applied to ERP studies in humans (e.g.^23^,^40^,^52^,^53^,^54^,^55^) and electrophysiological investigations in non-human primates (e.g.^2^,^56^,^57^). While this model has been criticised^58^ on the ground that it adds motor responses twice to the sum of the unisensory stimuli, with only one motor response corresponding to the AV condition (but see^40^), this criticism has been circumvented here by testing pupil dilation in the absence of overt motor response.

#### Correlation

If both RTs and pupil dilation reliably index multisensory processes a degree of correlation was expected such that faster RTs would be associated with larger pupil dilation in the multisensory condition and less so in the unisensory conditions. For this purpose, we computed a Pearson correlation (2-tailed) between these two measures for A, V and AV conditions.

## Additional Information

**How to cite this article**: Rigato, S. *et al.* Multisensory signalling enhances pupil dilation. *Sci. Rep.* **6**, 26188; doi: 10.1038/srep26188 (2016).

## Figures and Tables

**Table 1:** Multiple regression analyses for linear, quadratic, cubic and quartic effects of time of stimulus presentation predicting pupil dilation across 68 participants.

Variables	Auditory-Visual Stimuli	Auditory Stimuli	Visual Stimuli
	*β*	*β*	*β*
Time	0.14***	0.06***	0.06***
Time * Time	0.01	−0.01	−0.02
Time * Time * Time	−0.07***	−0.02*	−0.07***
Time * Time * Time * Time	−0.02	0.01	0.02

*Note. R*^*2*^’*s* for the total of shown effects in each model are 0.02, 0.01, and 0.01, respectively. Numbers are standardized regression coefficient, *β’s*. All effects are corrected for autocorrelation of data. Participants were treated as a random effect. *p < 0.05. ***p < 0.0001.
